# Polypharmacy and potentially inappropriate medication (PIM) use among older veterans with idiopathic pulmonary fibrosis (IPF) – a retrospective cohort study

**DOI:** 10.1186/s12890-025-03611-2

**Published:** 2025-04-21

**Authors:** Daniel M. Guidot, Marc Pepin, S. Nicole Hastings, Robert Tighe, Kenneth Schmader

**Affiliations:** 1 Geriatrics Research Education and Care Center, Durham VA Medical Center, 2035 Joshua Ln Durham, Durham, NC 27703 USA; 2 Division of Pulmonary and Critical Care, Duke University Medical Center, Durham, NC USA; 3 Division of Pulmonary, Durham VA Medical Center, Durham, NC USA; 4 Division of Geriatrics, Duke University Medical Center, Durham, NC USA; 5 Center of Innovation to Accelerate Discovery and Practice Transformation, Durham VA Health Care System, Durham Health Care System, Durham, NC USA

**Keywords:** Idiopathic pulmonary fibrosis (IPF), Older patients, Geriatrics, Polypharmacy, Potentially inappropriate medication (PIM), Health services

## Abstract

**Background:**

Idiopathic pulmonary fibrosis (IPF) is a deadly respiratory disease of older patients. IPF therapies (antifibrotics) are efficacious in slowing disease progression, but they are critically underutilized. Potential barriers to antifibrotic use are polypharmacy and potentially inappropriate medications (PIM). We examined the frequency of these factors for older patients with IPF.

**Methods:**

We retrospectively analyzed records of Veterans ≥ 65 years old in the Durham Veterans Affairs Health Care System who received a diagnosis of IPF and received care between 11 April 2023 and 9 September 2024. We analyzed medication profiles from the Corporate Data Warehouse including total medication counts, polypharmacy (≥ 5 medications), severe polypharmacy (> 15 medications), and prescription of a PIM in the anticholinergic, antidepressant, sedative, and antipsychotic classes using published geriatric guidelines (2023 Beers criteria, Screening Tool of Older People’s Potentially Inappropriate Prescriptions [STOPP] version 3). Identified PIMs underwent protocolized review to categorize them further as likely appropriate or inappropriate.

**Results:**

We identified 367 Veterans ≥ 65 years old with a diagnosis of IPF diagnostic during our study period. Total medication count was high for older Veterans (mean 14.2, SD 7.0). Veterans commonly had polypharmacy (350/367, 95.4%), severe polypharmacy (161/367, 43.9%), and ≥ 1 PIM (97/367, 26.4%). After protocolized review, 5.7% (21/367) of older Veterans with IPF had a likely inappropriate medication without documentation of a failed preferred alternative.

**Conclusion:**

For older Veterans with IPF, polypharmacy and PIM use were common and represent likely barriers to effective IPF pharmacotherapy initiation. Interventions that target these factors like deprescribing could improve antifibrotic use.

**Clinical trial number:**

Not applicable.

**Supplementary Information:**

The online version contains supplementary material available at 10.1186/s12890-025-03611-2.

## Background

Idiopathic pulmonary fibrosis (IPF) is a disease of progressive lung scar formation that leads to progressive respiratory failure, high morbidity, and poor survival [1]. IPF predominantly affects individuals over 65 years old, and in this group a diagnosis of IPF is associated with a median survival of just 3.8 years [1]. IPF is an important health concern for older Veterans. The Veteran population is enriched for IPF, and the median Veteran age at IPF diagnosis is 71 years old [2]. Fortunately, for both Veterans and other older Americans, medications exist that can slow IPF disease progression. Antifibrotics, the only FDA-approved class of medications for IPF, can improve survival and reduce the risk of IPF exacerbations requiring hospitalization [3]. Unfortunately, implementation of antifibrotic therapy in older patients with IPF remains low despite the benefits of therapy. In analysis of administrative claims data for private and Medicare Advantage plans, just 26.4% of patients with an IPF diagnosis were prescribed any antifibrotic; for Veterans, the rate was lower at 17%.^4^ While some factors have been implicated including socioeconomic status and geography [4], we do not know enough about the causes of low medication adoption for patients with IPF.

One likely barrier, understudied in IPF, is the role of polypharmacy and potentially inappropriate medication (PIM) use. Antifibrotics are beneficial medications but have high side effect profiles and interactions, and initiation of these therapies in older patients requires dedicated medication review and consideration [5, 6]. Polypharmacy, the use of multiple (often ≥ 5) medications, is a known risk factor for medication non-adherence in general for older patients [7]. In addition, older Veterans in the highest quartile of medication counts had 6.6-fold to 12.5-fold increased risk of medication-related problems including drug-drug or drug-disease interactions or taking medications at inappropriately high doses [8]. A PIM is a medication type, duration, or dosage that is known to have increased and potentially unacceptable risk in older adults, making the use of it in older adults often problematic. Lists of PIM classes and criteria commonly used in include the American Geriatrics Society’s Beers criteria [9] and the Pharmaceutical Care Network of Europe’s Screening Tool of Older People’s Potentially Inappropriate Prescriptions (STOPP) [10]. Studies in the US show that PIM use is associated a 3-fold increased risk of drug-related problems including significant central nervous system (CNS) and gastrointestinal (GI) side effect burdens. Thus, either polypharmacy or PIM use could prevent an older Veteran with IPF from initiating or adhering to a new and therapeutically important medication.

Medication factors like polypharmacy and PIM use are critical to understand for patients with IPF and their providers. Surveys of patients with IPF show that they report medication management as a key priority in their overall disease management [11]. Other studies have shown that antifibrotic medication adverse events and antifibrotic dose reductions are more common in older patients with IPF [12]. However, despite their importance, polypharmacy and PIM use in older Veterans with IPF are poorly defined. We sought to critically examine the medication profiles of Veterans diagnosed with IPF to determine the rate of polypharmacy and the presence of PIM use with a focus on inappropriate medications with a high clinical likelihood of preventing or impeding antifibrotic use.

## Methods

### Patient selection

We performed a retrospective analysis of Veterans ≥ 65 years old with IPF receiving care within the Durham VA Health Care System in North Carolina between April 11, 2023, and September 9, 2024. We identified our patient cohort utilizing the VA Corporate Data Warehouse (CDW). A Veteran Health Administration initiative, the CDW is a national integrated database of the electronic heath record data of all Veterans within the VA [13]. The VA Office of Information and Technology collects Veteran data from VA medical centers and consolidates them into a single, national data structure. These data for all Veterans are then made available to VA clinical innovators and researchers for use. From the CDW database, we identified all Veterans who were alive at the time of data collection (11 April 2024) and who were established with a Durham VA primary care provider. For this group, we collected and reviewed ICD-10 code entries linked to patient problem lists and patient clinical encounters during the study period. Adapting previously published methodology for identifying IPF cases within the VA health care system [2], we identified Veterans as having been diagnosed with IPF if they received a diagnosis of idiopathic pulmonary fibrosis by International Classification of Disease (ICD)-10 code and if they did not receive a diagnostic code for another competing form of ILD (see **Supplement**). For the final study cohort, we collected demographic and clinical information from the CDW.

### Medication data collection

For the final Veteran cohort, we also collected their active medication list recorded in the CDW database at the time of data collection (11 April 2024). This active medication list for each Veteran included all VA and recorded non-VA prescriptions in the health record on the date of data collection. This list included all medication names, dosages, and durations as well as the charted medication status (active, active/suspended, discontinued, or expired). We included in analysis all active medications and did not make any exclusions by indication, duration, or type. We included both daily and as needed/PRN medications; we also included oral medications, inhalers, and topical medications. Combination medications in one pill or prescription were counted once. Individual prescriptions of medications of the same drug type or class were all counted once. We excluded any medications with a medication status of discontinued or expired. We also excluded medication supplies and durable medical equipment. As a sensitivity analysis, we compared medication data collected from the CDW database against primary pharmacy data from the Durham VA pharmacy electronic health record.

### Polypharmacy and potentially inappropriate medication analysis

Medication counts were then analyzed for polypharmacy and for PIM presence. For polypharmacy, we defined medication profiles as meeting polypharmacy criteria if they were ≥ 5 and severe polypharmacy if they were > 15. For PIM presence, we compared the medications listed against two published guidelines for identifying PIMs: the 2023 Beers criteria [9] and the STOPP/START criteria, version 3 [10]. From these comprehensive lists of potentially inappropriate drug classes, we selected PIMs with either known major drug interactions (antiepileptics, sedatives) [14] or with overlapping side effect profiles (gastrointestinal symptoms, increased liver enzymes) [14]. Applying these criteria to the Beers and STOPP lists, we included the pharmacologic classes benzodiazepines, non-benzodiazepine sedatives, gabapentinoids, tricyclic antidepressants (TCA), non-TCA antidepressants, antipsychotics, and non-steroidal anti-inflammatory drugs. We excluded narcotic medication classes given their potential role in managing IPF symptoms like chronic cough [15]. We excluded proton pump inhibitors given their potential as a primary treatment in IPF and concomitant gastroesophageal reflux disease [16]. Non-steroidal anti-inflammatory medications (NSAIDs) were not included in the medication profiles of any Veterans with IPF and were not included. Medication classes that only apply to older patients with chronic kidney disease were not included.

All identified PIM medications for older Veterans with IPF then underwent a protocolized manual chart review. Adapting the STOPP guidelines, each PIM identified underwent a review of the PIM’s charted indication, dosage, and duration as well as documented tried preferred alternative medications in the same class for the same indication (see **Supplement**). After review, each identified PIM was then classified as likely appropriate or likely inappropriate.

Continuous data collected are presented as means (standard deviation or SD). Categorical variables are presented as count (percentage). Data were collected and analyzed using R version 4.2.2 and RStudio. The Durham Veterans Affairs (VA) Medical Center Institutional Review Board approved this study (IRB number #01882/001).

## Results

**Fig. 1:**
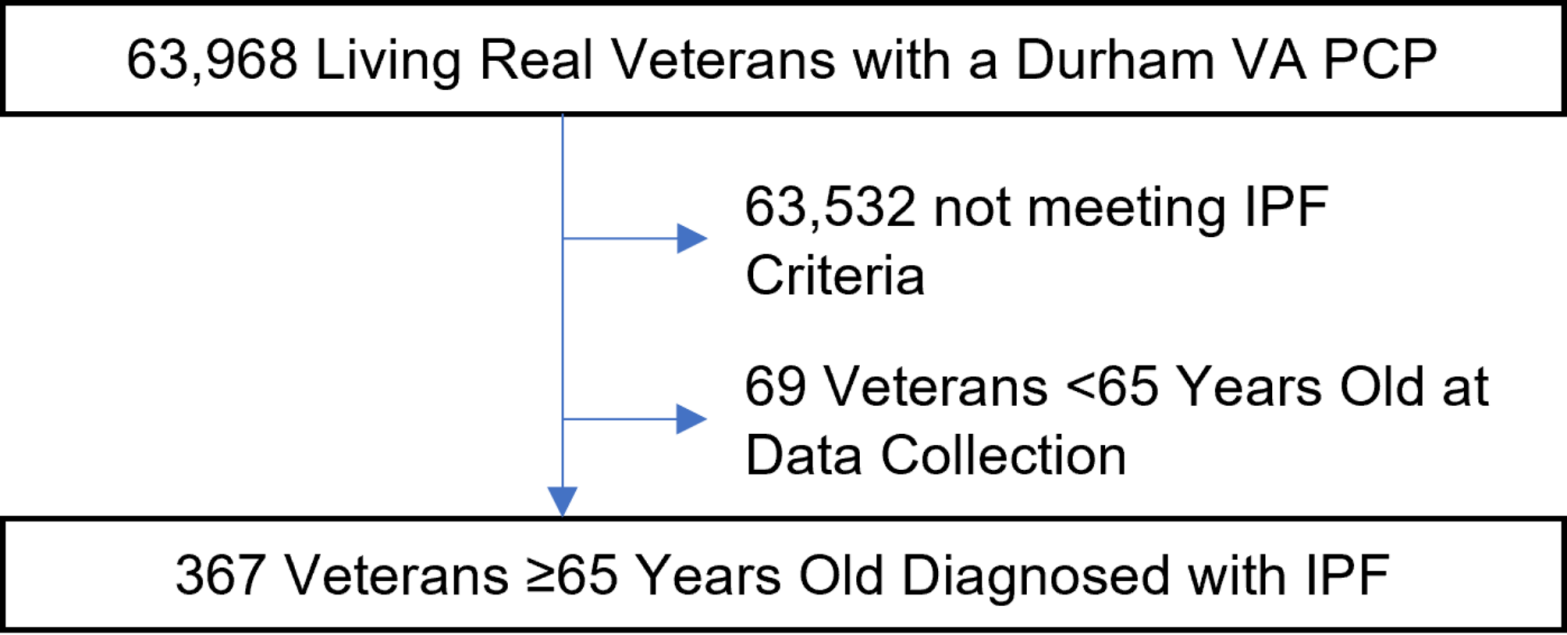
Consort diagram

We identified 367 Veterans ≥ 65 years old meeting IPF diagnostic criteria during our study period (Fig. 1). Veteran characteristics in Table 1. Older Veterans with IPF were most often male (351/367, 95.6%) and White (261/367, 71.1%). The number of active medications per Veteran was high (mean 14.2, SD 7.0). Polypharmacy (≥ 5 medications) and severe polypharmacy (> 15 medications) were common; the rate of polypharmacy was 95.4% (350/367) and the rate of severe polypharmacy was 43.9% (161/367). Conversely, the rate of antifibrotic medications of any type or dose frequency was low at 8.7% (32/367). Sensitivity analysis with primary Durham VA prescription data showed similar medication count totals compared with national VA data (see **Supplement**).

**Table 1:** Patient characteristics

Patient Characteristic	Veterans ≥ 65 Years Old with IPF *N* = 367
**Age at Data Pull**	
Mean (SD)	76.5 (6.5)
**Gender**	
Female	16 (4.4%)
Male	351 (95.6%)
**Race**	
Black or African American	89 (24.3%)
White	261 (71.1%)
Other/Declined/Unknown*	17 (4.6%)
**Number of Active Medications**	
Mean (SD)	14.2 (7.0)
**Polypharmacy (Count ≥ 5)**	
Yes	350 (95.4%)
No	17 (4.6%)
**Significant Polypharmacy (Count > 15)**	
Yes	161 (43.9%)
No	206 (56.1%)
**IPF Treatment (Antifibrotic)**	
Yes	32 (8.7%)
No	335 (91.3%)

IPF = idiopathic pulmonary fibrosis, SD = standard deviation, * Includes American Indian or Alaska Native, Asian, Multi-racial, Native Hawaiian or Other Pacific Islander.

Table 2 shows the medication profile analysis. In total 41.4% (152/367) of older Veterans with IPF had one or more medication in a PIM class with a clinical likelihood of interacting with an antifibrotic. Of the six PIM classes included in our analysis, 27.8% (102/367) of Veterans studied had an active medication in one PIM class, 10.6% (39/367) in two PIM classes, 2.5% (9/367) in three PIM classes, and 0.5% 92/367) in four PIM classes. The most common classes with active medications were non-TCA antidepressants (28.3%, 104/367) followed by gabapentinoids (21.3%, 78/367), TCA antidepressants (3.0%, 11/367), benzodiazepines (2.5%, 9/367), antipsychotics (2.2%, 8/367), and non-benzodiazepine sedatives/hypnotics (1.4%, 5/367). Table 3 shows the results of protocolized PIM review. Of the 152 Veterans with at least one active medication in a PIM class, protocolized review showed that 19.8% (26/131) of PIMs were classified as likely inappropriate (inappropriate type at an inappropriate dose or duration without any documentation of failure of a preferred alternative). This resulted in 7.1% (26/367) of all Veterans ≥ 65 with IPF having at least one medication that was deemed likely inappropriate. Of these inappropriate medications, the most common were tricyclic antidepressants (amitriptyline, doxepin, nortriptyline), gabapentin, selective serotonin reuptake inhibitors (SSRI) with higher side effect profiles (paroxetine), and chronic benzodiazepine prescriptions (lorazepam, diazepam, temazepam, see **Supplement**).

**Table 2:** Actively prescribed potentially and likely inappropriate medications for veterans over 65 at data collection

Medication Characteristic	Veterans ≥ 65 Years Old with IPF *N* = 367
Any PIM on Medication List*	
0	215 (58.6%)
≥1	152 (41.4%)
*Number of PIM Medication Classes*	
1	102 (27.8%)
2	39 (10.6%)
3	9 (2.5%)
4	2 (0.5%)
**Benzodiazepines**	
Yes	9 (2.5%)
No	358 (97.5%)
**Sedatives/Hypnotics (Non-Benzodiazepine)**	
Yes	5 (1.4%)
No	362 (98.6%)
**Gabapentinoids**	
Yes	78 (21.3%)
No	289 (78.7%)
**TCAs**	
Yes	11 (3.0%)
No	356 (97.0%)
**Other Antidepressants (Non-TCAs)**	
Yes	104 (28.3%)
No	263 (71.7%)
**Antipsychotics**	
Yes	8 (2.2%)
No	359 (97.8%)

* Included medications in classes outlined by Beers criteria or STOPP criteria including

PIM = potentially inappropriate medication, NSAID = non-steroidal anti-inflammatory drug, TCA = tricyclic antidepressant

**Table 3:** Potentially inappropriate medication (PIM) review and final classification

	Veterans ≥ 65 Years Old with IPF *N* = 367
**PIM**	
No	215 (58.6%)
Yes	152 (41.4%)
Likely Inappropriate Medication*	
No	341 (92.9%)
Yes	26 (7.1%)
*Number of Inappropriate Medications*	
1	23 (6.3%)
2	3 (0.8%)

*After protocolized manual review of the chart for medication against the STOPP criteria

## Discussion

Among Veterans ≥ 65 years old diagnosed with IPF, polypharmacy and PIM use were common and significant. In total, 95.4% had polypharmacy, 43.9% had severe polypharmacy, 41.4% had an active PIM, and 7.1% had a medication that is relatively contraindicated in older patients without evidence of appropriate indication or failure of preferred alternatives. The medications included in the PIM analysis were chosen for their competing toxicity profiles including gastrointestinal side effects that might complicate antifibrotic tolerance. This medication profile analysis of older Veterans with IPF showed both polypharmacy and PIM use have the potential to be significant barriers to effective therapy in IPF. These factors merit further investigation and could serve as targets for future health services interventions for Veterans with IPF.

Our study shows higher rates of polypharmacy than other reported populations of patients with IPF or fibrotic ILD. In a multi-site ILD registry study that included 645 patients with IPF, the rate of polypharmacy (≥ 5 medications) was reported at 53% [17] lower than the rate we found for our older Veteran population at 95.4%. Rates of polypharmacy in our study cohort are also lower than meta-analysis results for the general Veteran and active-duty military population (49%) [18]. The differences between our analysis and these other studies suggest several possible insights. There may be factors driving polypharmacy and PIM use independent of the diagnosis of IPF and are instead related to the challenges of care for patients with complex subspecialty conditions. Our reported rates were closer to rates reported in studies of older US patients with other complex chronic conditions like cancer (where polypharmacy was estimated at 84% and PIM use at 51%) [19]. Indeed, IPF has many similarities to cancer including complex treatments as well as high morbidity and mortality in older Veterans [19]. It is also notable that, for older patients with cancer, the addition of cancer-related therapy in the setting of polypharmacy and PIM use is associated with increased risk for adverse drug effects, drug-drug interactions, and non-adherence to cancer therapies [20–22]. There may also be Veteran-specific factors contributing to medication-related issues in our cohort. Factors such as increased prevalence of neuropsychiatric disorders like post-traumatic stress disorder (PTSD) could be implicated [23]. Finally, the age of our cohort could explain the higher rates of medication-related issues relative to other IPF cohorts given that polypharmacy is more common in older patients in the US [24]. Expanded future studies with relevant comparison populations and formal comparative analyses can help better understand the contributions of age-related factors, Veteran-specific factors, and disease-specific factors driving polypharmacy and PIM use in older Veterans with IPF.

The high rates of medication-related barriers stand in contrast to the low rates of antifibrotic use in our cohort and in national VA cohorts. In our cohort of older Veterans, the rate of antifibrotic prescription was 8.7%. This is compared with a rate of 17% for a national VA cohort of Veterans of all ages with a diagnosis of IPF [4] and compared with a rate of 26% for US Medicare claims analysis of adults over 65 outside the VA [25]. The national VA and US Medicare analyses demonstrate challenges in providing antifibrotic therapy both in Veteran and in older US populations with a diagnosis of IPF. Studies have shown that access to subspecialist expertise can impact effective and timely IPF diagnosis for Veterans [26], which may account for lower VA rates. Our cohort in particular might have lower antifibrotic rates that national VA cohorts because our population is exclusively over 65. The medication factors we identified in our study could serve as additional age-related barriers to care for Veterans. Other age-related factors including the impact of mobility, mentation, and patient preferences are not well described in older Veterans and merit further study.

Our study highlights a promising new target for developing strategies to address low treatment rates for Veterans with IPF. In addressing problems of polypharmacy and inappropriate medication use, deprescribing interventions have emerged as a promising strategy [27]. Deprescribing interventions use systematic medication review and application of relevant guidelines to identify inappropriate medication use and provide patients and providers recommendations for deprescribing them [27]. Analyses show that, in other older patient populations, deprescribing interventions have the potential to significantly reduce medication totals [28] and PIM use [29–31]. These interventions also have a track-record of success within the VA including the Durham VA. One such program is the Falls Assessment of Medications in the Elderly (FAME) Program [32], which identifies Veterans ≥ 65 with a flag for falls in their chart and a PIM in a category with high likelihood to increase fall risk (such as an anticholinergic medication). Identified Veterans then received a multidisciplinary medication review and deprescribing recommendation shared with Veterans and their providers. This program has high uptake among Veterans and providers and is associated with lower drug burden index measures and fewer new prescriptions drugs increasing fall risk [32]. Adapting this approach to Veterans with IPF at the Durham VA holds promise and merits evaluation. Multi-site or national analyses of Veteran populations could help determine the national rates of polypharmacy and PIM use in older Veterans with IPF and guide the design and implementation of national strategies to address them.

Strengths of our study include the breadth and scope of the VA electronic health record. We were able to pull pharmacy data from national sources and validate against primary Durham pharmacy electronic medication records. In addition, we further analyzed the medication profiles by examining PIM classes. Despite IPF being primarily a disease of older adults, prior medication evaluations of patients with IPF have not applied medication evaluation protocols for geriatric populations. Finally, our review applied a systematic medication review protocol derived from the STOPP criteria and Beers criteria to further quantify the number of likely inappropriate medications, identifying targets for Veterans including antidepressants with a high gastrointestinal side effect profile that would likely complicate or prevent antifibrotic initiation.

There are key limitations that need to be considered in this study. First, we applied a broad and comprehensive approach to quantifying medication counts that may differ from other approaches to evaluating medications. We included chronic scheduled oral medications but also inhalers, topical medicines, over-the-counter multivitamins or supplements, and as-needed prescriptions. Other analyses that focus only on oral medications or chronic medication use would yield different estimates. Another limitation is that the VA is comprehensive but might not capture all Veteran medications. Especially for older Veterans, dual-enrollment in both the VA and Medicare Part D plans is common [33]. For such individuals, the VA health record could miss active prescriptions provided by another provider. In addition, medications purchased without a prescription could be missed. This could explain the fact that no NSAID use was recorded for any older Veteran for IPF and that NSAID use could not be studied in our cohort. In addition, our analysis did not estimate the relative complexity of each medication identified. Depending on the frequency and route of administration of a medication, a single prescription could contribute differing degrees of overall medication burden to a patient. Another key limitation is that our medication analysis focused on the active medication profiles for these older Veterans with IPF at a single point in time only. This approach was pragmatic and allowed for analysis of a larger cohort; however, this approach does not allow for assessing changes in medication profiles over time. Frequent medication changes and intermittent short courses of medications could be missed by measuring a medication profile at a single point in time but still contribute significantly to medication burden. Analysis of dynamic medication profiles over time would be expected to find additional challenges for our cohort. Another limitation of note is that we applied Beers and STOPP criteria to a Veteran population, a group with distinct psychiatric comorbidities and other care needs related to a history of military service. We lack guidelines for classifying PIMs for this population in particular; determining specific recommendations for classifying PIMs among Veterans merits consideration by the VA geriatric research community. Finally, our PIM identification and medication review process was limited to a subset of PIM classes with a high clinical likelihood of interfering with IPF treatments. Thus, our analysis applied only a subset of the Beers and STOPP criteria that related to these medication classes. If we repeated analysis to include all PIM categories and all STOPP criteria, we would expect to find higher rates of PIM use and higher rates of likely inappropriate medication use for Veterans. While our analysis focuses on medication factors with a high likelihood of impacting IPF treatments, the full depth and complexity of polypharmacy for our cohort is likely even greater. There are likely opportunities for deprescribing in these Veterans completely independent of their IPF diagnosis or treatment considerations.

## Conclusion

For older Veterans with IPF, polypharmacy and PIM use were common with a subgroup of Veterans having a likely inappropriate drug in their active medication lists. In contrast to all this, rates of antifibrotic use were very low for Veterans ≥ 65 with IPF. Polypharmacy and PIM use merit further investigation as potential targets for clinical interventions to improve antifibrotic initiation and maintenance in older adults with IPF.

## Electronic supplementary material

Below is the link to the electronic supplementary material.

Supplementary Material 1

## Data Availability

Data for the results are housed within the Veterans Affairs Corporate Data Warehouse in Houston, Texas. Access requires VA approval to access; restrictions apply. Methods for extracting data from the Corporate Data Warehouse are available from the corresponding author on reasonable request.
