# Supplementary material for: Polypharmacy and potentially inappropriate medication (PIM) use among older veterans with idiopathic pulmonary fibrosis (IPF) – a retrospective cohort study
Source: BMC Pulm Med. 2025 Apr 21;25:186. doi: 10.1186/s12890-025-03611-2 (PMC12012958; doi:10.1186/s12890-025-03611-2)
Supplement: Supplementary file 1 — Supplementary Material 1 [file 12890_2025_3611_MOESM1_ESM.docx]

**Supplement**

**S1: IPF ICD Codes and Phenotype Algorithm**

| **ICD10Code** | **ICD10Description** |
| --- | --- |
| ***IPF*** |  |
| J84.17 | Other interstitial pulmonary diseases with fibrosis in diseases classified elsewhere |
| J84.170 | Interstitial lung disease with progressive fibrotic phenotype in diseases classified elsewhere |
| J84.178 | Other interstitial pulmonary diseases with fibrosis in diseases classified elsewhere |
| J84.10 | Pulmonary fibrosis, unspecified |
| J84.111 | Idiopathic interstitial pneumonia, not otherwise specified |
| J84.112 | Idiopathic pulmonary fibrosis |
| J84.17 | Other interstitial pulmonary diseases with fibrosis in diseases classified elsewhere |
| J84.170 | Interstitial lung disease with progressive fibrotic phenotype in diseases classified elsewhere |
| J84.178 | Other interstitial pulmonary diseases with fibrosis in diseases classified elsewhere |
| J84.89 | Other specified interstitial pulmonary diseases |
| J84.9 | Interstitial pulmonary disease, unspecified |
| ***Non-IPF ILD*** |  |
| C96.6 | Unifocal Langerhans-cell histiocytosis |
| D86.0 | Sarcoidosis of lung |
| D86.2 | Sarcoidosis of lung with sarcoidosis of lymph nodes |
| D86.9 | Sarcoidosis, unspecified |
| E75.21 | Fabry (-Anderson) disease |
| E75.22 | Gaucher disease |
| E85.9 | Amyloidosis, unspecified |
| J60. | Coalworker's pneumoconiosis |
| J61. | Pneumoconiosis due to asbestos and other mineral fibers |
| J62.8 | Pneumoconiosis due to other dust containing silica |
| J63.0 | Aluminosis (of lung) |
| J63.1 | Bauxite fibrosis (of lung) |
| J63.2 | Berylliosis |
| J63.3 | Graphite fibrosis (of lung) |
| J63.4 | Siderosis |
| J63.5 | Stannosis |
| J63.6 | Pneumoconiosis due to other specified inorganic dusts |
| J64. | Unspecified pneumoconiosis |
| J66.8 | Airway disease due to other specific organic dusts |
| J67.0 | Farmer's lung |
| J67.1 | Bagassosis |
| J67.2 | Bird fancier's lung |
| J67.3 | Suberosis |
| J67.4 | Maltworker's lung |
| J67.5 | Mushroom-worker's lung |
| J67.6 | Maple-bark-stripper's lung |
| J67.7 | Air conditioner and humidifier lung |
| J67.8 | Hypersensitivity pneumonitis due to other organic dusts |
| J67.9 | Hypersensitivity pneumonitis due to unspecified organic dust |
| J68.4 | Chronic respiratory conditions due to chemicals, gases, fumes and vapors |
| J70.1 | Chronic and other pulmonary manifestations due to radiation |
| J70.8 | Respiratory conditions due to other specified external agents |
| J82. | Pulmonary eosinophilia, not elsewhere classified |
| J82.81 | Chronic eosinophilic pneumonia |
| J82.82 | Acute eosinophilic pneumonia |
| J82.83 | Eosinophilic asthma |
| J82.89 | Other pulmonary eosinophilia, not elsewhere classified |
| J84.01 | Alveolar proteinosis |
| J84.02 | Pulmonary alveolar microlithiasis |
| J84.03 | Idiopathic pulmonary hemosiderosis |
| J84.09 | Other alveolar and parieto-alveolar conditions |
| J84.113 | Idiopathic non-specific interstitial pneumonitis |
| J84.114 | Acute interstitial pneumonitis |
| J84.115 | Respiratory bronchiolitis interstitial lung disease |
| J84.116 | Cryptogenic organizing pneumonia |
| J84.117 | Desquamative interstitial pneumonia |
| J84.2 | Lymphoid interstitial pneumonia |
| J99. | Respiratory disorders in diseases classified elsewhere |
| K50.00 | Crohn's disease of small intestine without complications |
| K50.10 | Crohn's disease of large intestine without complications |
| K50.80 | Crohn's disease of both small and large intestine without complications |
| K50.90 | Crohn's disease, unspecified, without complications |
| M05.10 | Rheumatoid lung disease with rheumatoid arthritis of unspecified site |
| M30.1 | Polyarteritis with lung involvement [Churg-Strauss] |
| M31.0 | Hypersensitivity angiitis |
| M31.30 | Wegener's granulomatosis without renal involvement |
| M31.7 | Microscopic polyangiitis |
| M32.10 | Systemic lupus erythematosus, organ or system involvement unspecified |
| M33.20 | Polymyositis, organ involvement unspecified |
| M33.90 | Dermatopolymyositis, unspecified, organ involvement unspecified |
| M34.0 | Progressive systemic sclerosis |
| M34.81 | Systemic sclerosis with lung involvement |
| M35.00 | Sicca syndrome, unspecified |
| M35.00 | Sjogren syndrome, unspecified |
| M45.9 | Ankylosing spondylitis of unspecified sites in spine |
| Q85.00 | Neurofibromatosis, unspecified |
| Q85.1 | Tuberous sclerosis |

- Patients were defined as meeting IPF criteria if they had one or more IPF codes and no competing non-IPF ILD codes entered during the study period.

**S2: Criteria for Determining Whether a PIM was Likely Inappropriate**

PIM Classes Identified (by Beers or STOPP criteria for PIM):

- Antidepressant
- Benzodiazepine
- Non-benzodiazepine hypnotic (zolpidem)
- Antipsychotic
- Gabapentinoid
- Non-steroidal anti-inflammatory drug (none identified)

Antidepressant PIM classified as inappropriate if (any of the below):

- Any type prescribed without an indication
- Any duplicated drug in same class (two simultaneous SSRIs, SNRIs, or TCAs)
- TCA-specific contraindications:
  - TCA in a patient with dementia, glaucoma, falls, cardiac conduction problems
  - TCA ss first line for depression (no prior failed preferred alternative)
- SNRI-specific contraindications
  - SNRI in a patient with severe hypertension
- SSRI-specific contraindications
  - SSRI in a patient with chronic hyponatremia (Na < 130), recent severe bleeding
  - Duloxetine in a patient with urinary urgency

Benzodiazepine PIM classified as inappropriate if (any of the below):

- Benzodiazepine prescribed for >4 weeks for any indication
- Benzodiazepines in a patient with setting of chronic respiratory failure or falls

Non-Benzodiazepine PIM classified as inappropriate if (any of the below):

- Non-benzodiazepine hypnotic drugs (i.e. zolpidem) in a history of falls

Antipsychotic PIM classified as inappropriate if (any of the below):

- Antipsychotics in a patient with parkinsonism or lewy body dementia (not quetiapine or clozapine)
- Antipsychotics for behavioral symptoms in a patient with dementia

Gabapentinoid PIM classified as inappropriate if (any of the below):

- Gabapentinoid prescribed for non-neuropathic pain

**S3: Comparison of Primary Pharmacy Data Pull and Durham Pharmacy Record**

|  | <65 (N=71) | 65+ (N=367) |
| --- | --- | --- |
| **Number of Active Prescriptions** |  |  |
| Mean (SD) | 13.8 (6.77) | 14.2 (7.00) |
| Median (Q1, Q3) | 12.0 (8.50, 18.0) | 14.0 (9.00, 18.0) |
| Range | [2.00, 34.0] | [0, 55.0] |
| **Number of Active Prescriptions (Durham VA Pharmacy Check)** |  |  |
| Mean (SD) | 13.4 (4.76) | 14.3 (6.21) |
| Median (Q1, Q3) | 13.0 (9.00, 16.5) | 14.0 (10.0, 18.0) |
| Range | [6.00, 24.0] | [3.00, 30.0] |
| Missing | 44 (62.0%) | 182 (49.6%) |

**S5: Likely Inappropriate Medication Type and Frequency**

| Medication | Frequency |
| --- | --- |
| Amitriptyline | 4 |
| Clonazepam | 1 |
| Doxepin | 2 |
| Gabapentin | 4 |
| Lorazepam | 3 |
| Nortriptyline | 5 |
| Paroxetine | 6 |
| Pregabalin | 1 |
| Temazepam | 2 |
| Zolpidem | 1 |

Frequency includes the total number of cases with each medication.
